# Critical Examination of Distance-Gain-Size (DGS) Diagrams of Ultrasonic NDE with Sound Field Calculations

**DOI:** 10.3390/s23157004

**Published:** 2023-08-07

**Authors:** Kanji Ono, Hang Su

**Affiliations:** 1 Department of Materials Science and Engineering, University of California, Los Angeles, CA 90095, USA; 2 Department of Civil and Environmental Engineering, University of California, Los Angeles, CA 90095, USA; hangsu2018@g.ucla.edu

**Keywords:** Distance-Gain-Size (DGS) diagrams, ultrasonic non-destructive evaluation, sound fields, ultrasonic transducer, backward diffraction path, two-way diffraction

## Abstract

Ultrasonic non-destructive evaluation, which has been used widely, can detect and size critical flaws in structures. Advances in sound field calculations can further improve its effectiveness. Two calculation methods were used to characterize the relevant sound fields of an ultrasonic transducer and the results were applied to construct and evaluate Distance-Gain-Size (DGS) diagrams, which are useful in flaw sizing. Two published DGS diagrams were found to be deficient because the backward diffraction path was overly simplified and the third one included an arbitrary procedure. Newly constructed DGS diagrams exhibited transducer size dependence, revealing another deficiency in the existing DGS diagrams. However, the extent of the present calculations must be expanded to provide a catalog of DGS diagrams to cover a wide range of practical needs. Details of the new construction method are presented, incorporating two-way diffraction procedures.

## 1. Introduction

Methods of ultrasonic non-destructive evaluation (UNDE) have played key roles in providing quality assurance and failure prevention in many industries [1,2,3,4]. With increased uses of phased-array probes, the importance of sound field simulation has been recognized, and modeling tools have been developed and implemented [4,5]. The basic sound field modeling of a circular disc ultrasonic transducer has been studied for many years [6,7,8,9,10,11,12,13,14,15,16,17,18,19,20,21,22,23,24,25,26,27], and its theoretical basis was established decades ago. Sound pressure profiles of a circular or rectangular disc can be readily plotted using a MATLAB program (e.g., [28,29]). Similar programs can also be written using tools from open software platforms (e.g., [4,30]).

**Figure 1:**
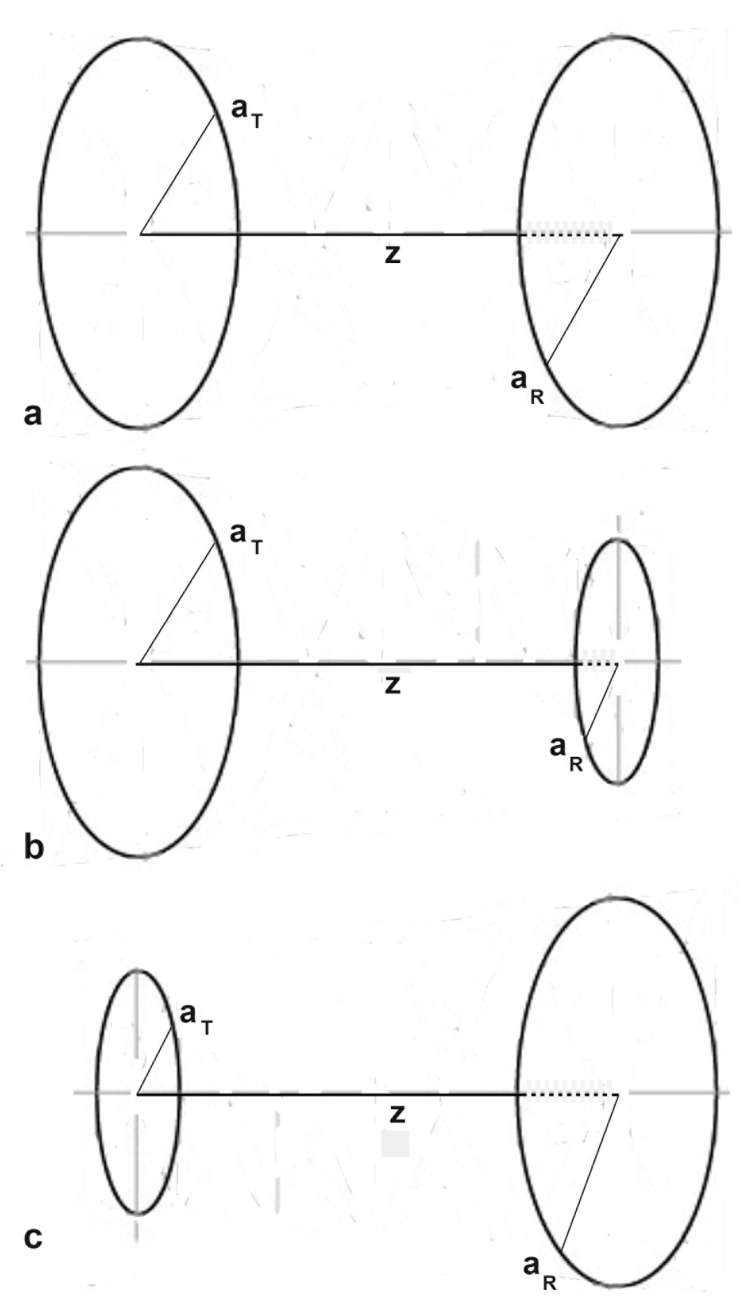
Coaxial transmitter–receiver arrangements. (**a**) Equal-sized transmitter–receiver pair, **S** = 1. (**b**) Small receiver case, **S** < 1. (**c**) Large receiver case, **S** > 1.

The initial uses of sound field calculations were to correct diffraction losses in the attenuation measurements of ultrasonic waves in the region close to a transmitter or the near-field zone [11,13,31]. This was greatly simplified when Roger and van Buren [22] derived an analytical expression for diffraction losses with a pair of coaxial circular transmitter and receiver of identical radii, **a**. This arrangement is shown in Figure 1a. The diffraction loss, **D**, is given by

**D** = {[cos(2π/**Z**) − J_0_(2π/**Z**)]^2^ + [sin(2π/**Z**) − J_1_(2π/**Z**)]^2^}^0.5^, (1)

where **Z** = **z v/f a^2^**, where **v** is the wave velocity, **z** is the propagation distance, and **f** is the frequency, respectively. Earlier in 1966, the same equation for diffraction losses was derived by Torikai [15] from the Lommel integral using Bessel function identities [7,8,9]. Two different derivation methods confirmed the validity of Equation (1) or the Torikai–Roger–van Buren (TRvB) equation. This equation has been extensively used in ultrasonic attenuation studies [31,32,33,34,35]. Using the near-field distance, **N** = **f a^2^**/**v**, the **Z** parameter can be given as **Z** = **z**/**N** (i.e., the wave propagation distance in the unit of the near-field distance). In turn, this implies that the TRvB equation has no direct dependence on the transducer radius, **a**, wavelength, **λ** = **v**/**f**, or their ratio, **a/λ**. In usual attenuation studies, **z**, **v**, and **a** are fixed, and **D** is obtained as a function of **f**.

Another application of sound field calculations is to determine the size of a flaw or discontinuity (usually modeled by a circular reflector) in pulse-echo ultrasonic testing (PEUT). In 1958, Krautkramer [36,37] constructed a Distance-Gain-Size (DGS) diagram from the early knowledge of sound field calculations and laboratory tests with some assumptions of ultrasound behavior. Key features of his original construction of DGS diagrams are still retained in the current DGS diagrams [1,3,5]. Many subsequent studies have refined DGS diagrams [38,39,40,41,42,43,44,45,46,47,48], and an ISO standard was issued in 2012 [49]. Unfortunately, the scientific foundation of the published DGS diagrams has remained obscure due to non-disclosure of their construction methods. In a conference publication, Mundry [38,39] reported on sound field calculations, but only an equation containing four integrals was presented. He stated that the integrals were solved using Simpson’s method, giving some echo amplitude distribution curves. A DGS diagram was also published, but without describing the procedures to compute the DGS diagram. Kimura [41] calculated the sound field using Torikai formulas [15,16] and presented DGS diagrams. Again, no description of the DGS construction was provided. In ISO16811 [49], another DGS diagram was published without any reference. This diagram was called a “general” DGS diagram, which was the term first used by Krautkramer [1], and these two appear to be related in some ways. Kleinert [5] presented various applications of DGS diagrams from ISO16811, but again, without references on the origin of the general DGS diagram. More recent modeling studies [27,42,43,44,45,46,47,48] have established the mathematical foundation of DGS calculations, but these were not extended to actually compute and replace earlier DGS diagrams.

In terms of diffraction theories, PEUT is considered to be two-way diffraction [27], which consists of the transmitter-to-reflector (forward) path and reflector-to-receiver (backward) path. Each path has different transmitter and receiver sizes, which correspond to different values of **N**. Figure 1 shows three possible transmitter–receiver combinations. Taking **a_T_** and **a_R_** as the transmitter and receiver radii, **S** is defined as **S** = **a_R_/a_T_**. For the three cases in Figure 1a–c, we have **S** = 1, **S** < 1, and **S** > 1, respectively. Two separate sound field calculations are needed for each complete (or round-trip) sound path, combined on the basis of physical propagation distances [38]. Thus, it is necessary to appraise the methods used to construct DGS diagrams and to reconstruct them on the basis of two-way diffraction methods. However, Daly and Rao [27] used the reciprocity theorem, applicable only to a point source–receiver pair, making their model inappropriate for PEUT cases that rely on transducers of finite sizes. Furthermore, the original Krautkramer construction [37,38] indeed embodied the two-way diffraction concept. From the initial DGS diagram of Krautkramer to the present, the available DGS diagrams have been incorporated to industrial PEUT and its more advanced test procedures (e.g., [1,48,49,50]). However, their origins have not been clarified in the UNDE literature, even though sound field calculations have been refined over several decades [6,7,8,9,10,11,12,13,14,15,16,17,18,19,20,21,22,23,24,25,26,27,28,29,30].

In this study, the interrelation between the DGS diagrams and sound field calculations was explored. First, the available sound field calculations by Seki, Yamada, and Khimunin [13,17,18] as well as the TRvB equation [15,22] were examined, followed by Torikai’s analytical expressions of the sound field of a circular transmitter [15,16]. It is essential to use the Torikai expressions because these cover both the inside and outside regions in front of a transmitter. These calculations were compared with the MATLAB calculations based on the Zemanek model [19,28]. In this case, the transmitter diameter was varied from 0.8 to 50.8 mm in order to cover the range of typical transducers used in PEUT. These also characterized the reflection from a circular flaw of various sizes for the backward path in PEUT. The construction methods of earlier DGS diagrams by Mundry, Kimura, and the general DGS diagram in ISO16811 were explored next. These were then compared to the two-way diffraction models of DGS diagrams based on the sound field calculations. Discussion of the results, conclusions, and recommendations are given.

## 2. Sound Field Calculations

### 2.1. Results of Previous Studies

**Figure 2:**
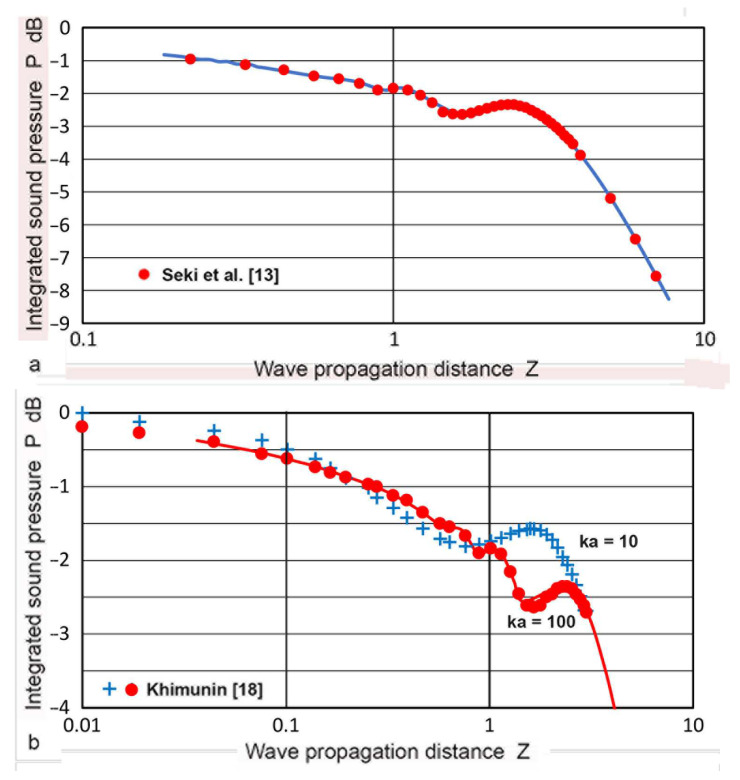

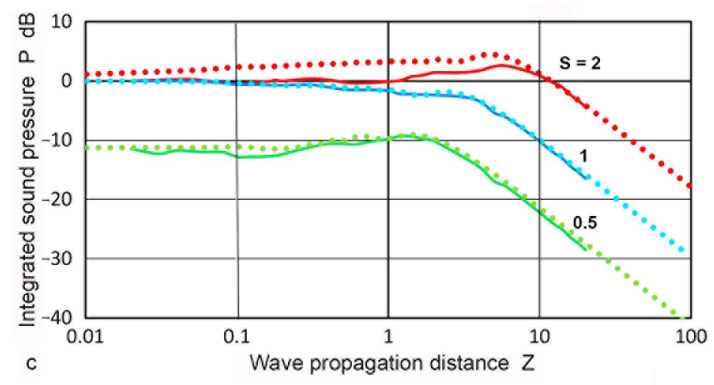
(**a**) Integrated sound pressure, **P**(**Z**) vs. **Z** (red dots) from Seki et al. [13]. The diffraction loss, **D**, from Equation (1) is also plotted (blue curve). (**b**) **P**(**Z**) vs. **Z** (blue **+**) from Khimunin [18] and **D** (red curve). (**c**) **P**(**Z**) vs. **Z** from Yamada and Fujii [17] (solid curves in green, **S** = 0.5, in blue, **S** = 1, in red, **S** = 2) and from the Torikai equations (dotted curves, same color code; see text).

Seki et al. [13] made the first numerical calculation of diffraction loss applicable to PEUT. In this case, the transmitter and a receiver were of the same size and placed coaxially at **z**, as shown in Figure 1a. The sound pressure was integrated over the face of the receiver. Tabulated root-mean-square (rms) values of integrated sound pressure, **P**(**Z**), vs. **Z** were given for **ka** values of 50 to 1000. Here, **Z** = **z/N** and wavenumber, **k** = 2π/**λ**. In Figure 2a, changes in **P**(**Z**) values at **ka** = 50, given in red dots, were compared to the diffraction loss, **D**, from the TRvB equation, matching **P**(1) to **D**(1). The **P** and **D** values agreed well with each other. The **P** values for larger **ka** values were essentially identical to those at **ka** = 50. This behavior is in agreement with the **ka**-independence of the TRvB equation. Khimunin [18] provided the exact calculations of **P** for **ka** of 10 to 1000. The **P** values showed less than a 0.5 dB difference at a given **Z** value, but differences reached a maximum of 1.15 dB at **Z** = 1.4. The difference was typically less than 0.1 dB, implying **ka**-independence for larger **Z**. A comparison of his **P** values (for **ka** = 10 in blue + symbols and 100 in red dots) to the TRvB equation (drawn in red) is shown in Figure 2b. Again, a good agreement was shown for **ka** = 100, but differences were present for low ka values at **Z** ≤ 3. The range of Z plotted was from 0.01 to 3, entering into the far-field region.

When the transmitter and a receiver are of different sizes and are placed coaxially at **z**, as shown in Figure 1b,c, the area of integration has to be changed. Such **P**(**Z**) calculations were first given by Yamada and Fujii [17] for the half- and double-sized receivers (i.e., **S** = 0.5 and **S** = 2). Yamada and Fujii started from the Lommel formulas and obtained three integral expressions for **S** ≤ 1, **S** > 1, and **S** = 0 with integrands composed of exponential and trigonometric functions. Results of the **P**(**Z**) values for **S** = 0.5, 1, and 2 were given graphically (in green, blue, and red curves). Their **P**(**Z**) are plotted in Figure 2c along with the Torikai calculations (in dotted curves of same color), as presented below. Note that the results of Yamada and Fujii included the integration constants, requiring amplitude-level adjustments. Because areal ratios are 0.25 and 4, the far-field **P**(**Z**) values are expected to differ by 12 dB from the unity **S** case.

### 2.2. Torikai Formulation

Torikai developed another approach for calculating the sound pressure, **p**, at a point (**X**,**Z**) or **p**(**X**,**Z**) using **X** = **x/a_T_**. Here, **p** represents the sound-pressure value at a single point, (**X**,**Z**). He started from the Lommel formulas as in Seki, producing the following equations that included Bessel function series:

(2) $$p=1-\exp(-i\pi (1+X^{2})/Z)\sum_{n=0}^{\infty }{\left(iY\right)}^{n}•J_{n}(2\pi Y/Z),\;\mathrm{for}\;X\;\leq \;1$$

(3) $$p=1-\exp(-i\pi (1+X^{2})/Z)\sum_{n=1}^{\infty }{\left(i/Y\right)}^{n}•J_{n}(2\pi Y/Z),\;\mathrm{for}\;1\;\leq \;X$$

Two special-case solutions are

**p** = 1 − cos(π/**Z**) + *i* sin(π/**Z**), for **X** = 0 (4)

**p** = [1 − cos(2π/**Z**)•J_0_(2π/**Z**) + *i* sin(2π/**Z**)•J_1_(2π/**Z**)]/2, for **X** = 1, (5)

where *i*^2^ = –1, and J_0_ and J_n_ are the Bessel function of the first kind of order 0 and **n**. The use of the Lommel formulas limits the value of **z** = **NZ** to be larger than the transmitter radius, **a_T_** [15,16]. This Lommel limit typically excludes the near-field region, although all the results will be shown in order to compare the two calculation methods examined in this study.

The Torikai equations can readily be computed using an Excel worksheet by providing an **X** value and a set of **Z** values, yielding a set of **p** values for given **Z**s (in columns) at the given **X** value. This is repeated for each **X** value. In the computation presented below, **Z** was from 0.001 to 100 and the order, **n**, was set to 20, but no difference in results was found by using **n** = 12. Repeating the same computation for the systematically varied **X** values, a table can be constructed for the **p**(**X**,**Z**) values. The **X** values were incremented at ∆**X** = 0.01 for **X** ≤ 0.1, increasing to ∆**X** = 0.05 for 0.1 < **X** < 8. This tabulation was followed by numerical integration utilizing the central circle and concentric ring areas.

**Figure 3:**
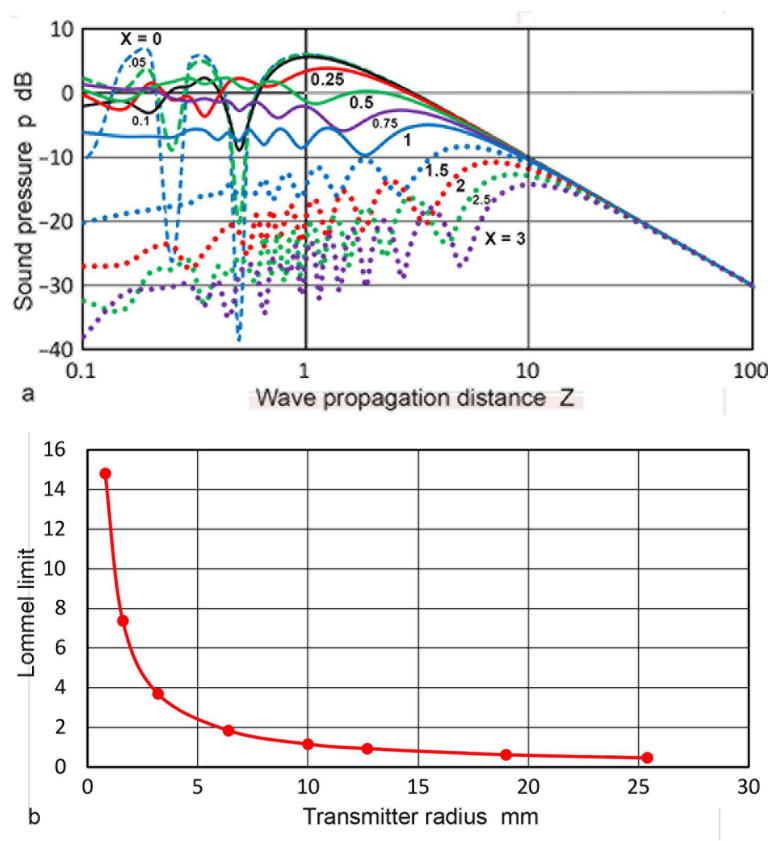
(**a**) Sound pressure, **p**(**X**,**Z**) vs. **Z** for various **X** values. **X** = 0: blue dash curve, 0.05: green dash, 0.1: black, 0.25: red, 0.5: green, 0.75: purple, 1.0: blue, 1.5: blue dot, 2.0: red dot, 2.5: green dot, 3.0: purple dot. (**b**) Lommel limit in **Z** vs. transmitter radius in mm.

The representative variation of **p**(**X**,**Z**) against **Z** is plotted in Figure 3a for the **X** values of 0 to 3. The on-axis (**X** = 0) behavior of **p** followed the well-known theoretical relation and showed many dips at **Z** below 0.01 [1,2,51]. Three dips are visible in Figure 3a and the depth at **Z** = 0.5 reached −38 dB. At least 100 more dips exist for **Z** between 0.1 and 0.00001 when calculations are made using a finer step in **Z**. For the three cases of **X** ≤0.1, sharp dips in **p** occurred at **Z** < 1. The dips resulted from diffraction effects, similar to the case of **X** = 0, represented by Equation (4). For 0.25 ≤ **X** ≤ 1, the **p** values were relatively unchanged with minor fluctuations in the region of **Z** = 2 to 7. For **X** > 1, a general trend was the reduction in oscillations toward low **Z** after deviating from the inverse **Z** relation in the far-field region. These are shown by the dotted curves. These **p**-variations outside the transmitter face were expected as sideway spreading of the sound fields increased as **Z** increased. In all cases, the **p–Z** slope became −1 in the far-field region, as expected from the spherical wave behavior. The Lommel limit for the above calculation is given by **Z** = **a_T_**/**N**. Taking the wavelength of 5.9 mm corresponding to that of steel at 1 MHz, the limiting **Z** values were plotted against **a_T_** in Figure 3b. For larger **a_T_** values, the limiting **Z** values were 0.5 to 1, while it was 15 for the smallest examined, or **a_T_** = 0.4 mm.

**Figure 4:**
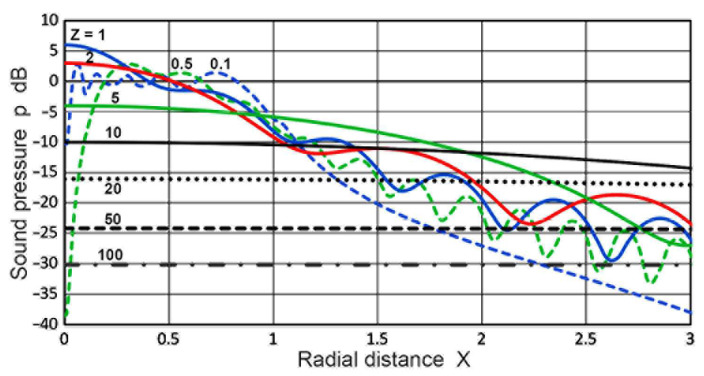
Sound pressure, **p**(**X**,**Z**) vs. normalized radius, **X**, at various values of **Z**. **Z** = 0.1: blue dash, 0.5: green dash, 1.0: blue, 2.0: red, 5.0: green, 10: black, 20: black dot, 50: black dash, 100: black dash-dot.

In Figure 4, changes in **p**(**X**,**Z**) against **X** were plotted for **X** values of 0 to 3. In the near-field region of **Z** of 0.1 or 0.5 (blue or green dash curve), the **p** values fluctuated by 13 or 43 dB, decreasing by 14 or 10 dB as **X** went from 0 to 1 (at the edge of the transmitter). For a **Z** of 1 or 2 (blue or red curve), the **p** values varied smoothly for low **X** and gradually decreased as **X** increased with oscillations. At **Z** = 5 (green curve), the initial **p** value at **X** = 0 decreased 10 or 6 dB from the blue or red curve, but this decrease in **p** became smaller and more gradual to **X** = 0.9. For **X** ≥ 1, its magnitude exceeded those for lower **Z**. As **Z** increased to 10 or above, well into the far-field region, the **p** values were less affected by **X**, becoming essentially independent of **X** at **Z** ≥ 20 for **X** < 3. At **Z** = 50 and 100, the **p** values decreased by 1 and 0.25 dB from **X** = 0 to **X** = 8.

**Figure 5:**
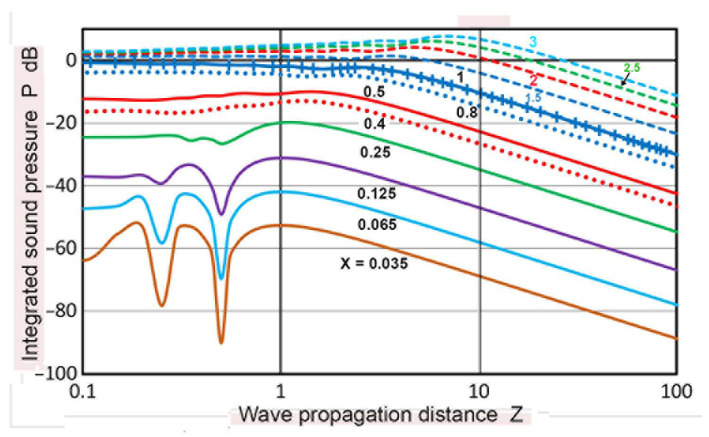
Integrated sound pressure, **P**(**X′**,**Z**) vs. **Z**. **X′** = 0.035: brown curve, 0.065: light blue, 0.125: purple, 0.25: green, 0.4: red dot, 0.5: red, 0.8: blue dot, 1.0: blue, 1.5: blue dash, 2: red dash, 2.5: green dash, 3.0: light blue dash. **D**(**Z**) vs. **Z** in blue **+**.

**Figure 6:**
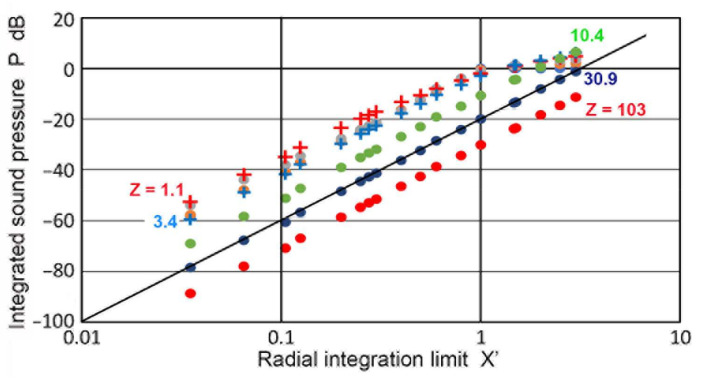
Integrated sound pressure, **P**(**X′**,**Z**) vs. radial integration limit, **X′**, for different **Z** values. **Z** = 0.1: orange dot, 0.3: gray dot, 1.1: red **+**, 3.4: blue **+**, 10.4: green dot, 30.9: blue dot, 103: red dot. Blue line through blue dots represents **X′**^2^ or the slope = 2.

Using sectional integration, integrated values of **p** or **P**(**X′**,**Z**) were determined for **X′** of 0.035 to 3.0. Note that **X′** represents the limit of areal integration. Plots of **P** vs. **Z** are given in Figure 5. Additionally plotted with **+** symbols are **D** values from the TRvB equation, which correspond to the case of **P**(1,**Z**). These were matched at **Z** = 1 by subtracting a constant (10.34 dB) from the **P** values in dB. All of the other **P** values were also corrected by using the same constant. The **P–Z** curve for **X′** = 1 and the **D**-values agreed within 0.026 dB on average for 0.1 ≤ **Z** ≤ 100, and the maximum difference was 0.21 dB. The calculated **P–Z** curves were mostly smooth, but dips of 3 to 30 dB occurred for **X′** ≤ 0.2 at **Z** = 0.5. These dips reflected the presence of dips in the **p** vs. **Z** curves shown in Figure 3a. Even with integration over a non-zero area ahead of the transmitter, the variation of **P** values was not smoothed out, producing the dips. Note that the **P–Z** curves for **X′** > 1 initially increased until the far-field region initiated. In contrast, those for **X′** ≤ 1 showed a nearly flat region (with peaks and dips at lower **X′**s) before the far-field region began. In the far-field region, **P**(**X′**,**Z**) decreased with 1/**Z**, as was the case of **p**(**X**,**Z**). Because of the averaging effects, most of the sharp fluctuations in the **p** values found in Figure 3a disappeared in the **P–Z** plots (Figure 5). In the far-field region (**Z** ≥ 10), **P** values at a constant **Z** increased with **X′**^2^, as shown in Figure 6. This **P**–**X′**^2^ relation persisted to **X′** of 3. For the lower **Z** of 0.1 to 3.4, the same areal dependence was again found, but the slope started to decrease above an **X′** of 0.3, clearly flattening upon reaching **X′** = 1. This indicates that, in front of the transmitter, the distribution of the integrated sound pressure is uniform, even in the near-field. In light of the widely varying sound fields in the near-field, as shown in Figure 3a and Figure 4, this was surprising, but the averaging effects through integration procedures reduced the fluctuations due to diffraction.

The above calculation of **P**(**X′**,**Z**) can be modified to obtain **P**(**X′**,**f**) for a fixed **z** value. **P**(**X′**,**f**) is equivalent to **D** from the TRvB equation, except now the values of **a_T_** and **a_R_** can be different. To obtain **D** equivalent values, one needs to calculate **Z** for a single **z** value with a sequence of frequency, **f**, using **Z** = **z v**/**f a_T_^2^**. With the identical Excel setup, **P**(**X′**,**f**) can be calculated for the frequency sequence, which is dictated by the fast Fourier transform (FFT) being used. Using the **P**(**X′**,**f**) sequence, ultrasonic attenuation can be measured even when **a_T_** and **a_R_** are unequal. This is more cumbersome than the use of the TRvB equation, but does offer an approach to obtain ultrasonic attenuation when only an unequal transducer pair is available.

In the Torikai and the following Zemanek calculations, transmitters were treated to produce a uniform displacement output over the entire face. This was experimentally verified on several ultrasonic transducers [52].

### 2.3. Sound Field Simulation Using Zemanek Model

Another method to calculate the sound pressure relies on computer software based on a theoretical model. In this study, a MATLAB program by Gasparini [28] was used that is based on a model by Zemanek [17]. This model allows for sound field computation without introducing a geometrical approximation, which was included in the Lommel formulas. A minor change in the output routine of the Gasparini program produced a table of sound pressure, **p**, as a function of **X** and **Z′** = **z**/**λ**. Specifically, the input parameters were the initial values and step increments of **X** and **Z′**. Additionally, the transmitter diameter, 2**a_T_**, and wavelength, **λ**, were inserted. Calculations were performed for 2**a_T_** values of 0.8, 1.0, 1.6, 3.2, 6.4, 10, 12.7, 19, 25.4, 38, and 50.8 mm with **λ** = 5.9 mm. The **X** step was 0.2 mm for all diameter values. For the four largest diameters, an **X** step of 0.4 mm was also used, while an **X** step of 0.1 mm was used for transmitter diameters of 0.8 and 1.0 mm. The **Z′** step was 0.01 up to **Z′** = 10 and 2 for **Z′** = 10 to 96. In these calculations, the transmitter radius to wavelength ratio (**a_T_/λ**) varied from 0.068 to 4.37.

**Figure 7:**
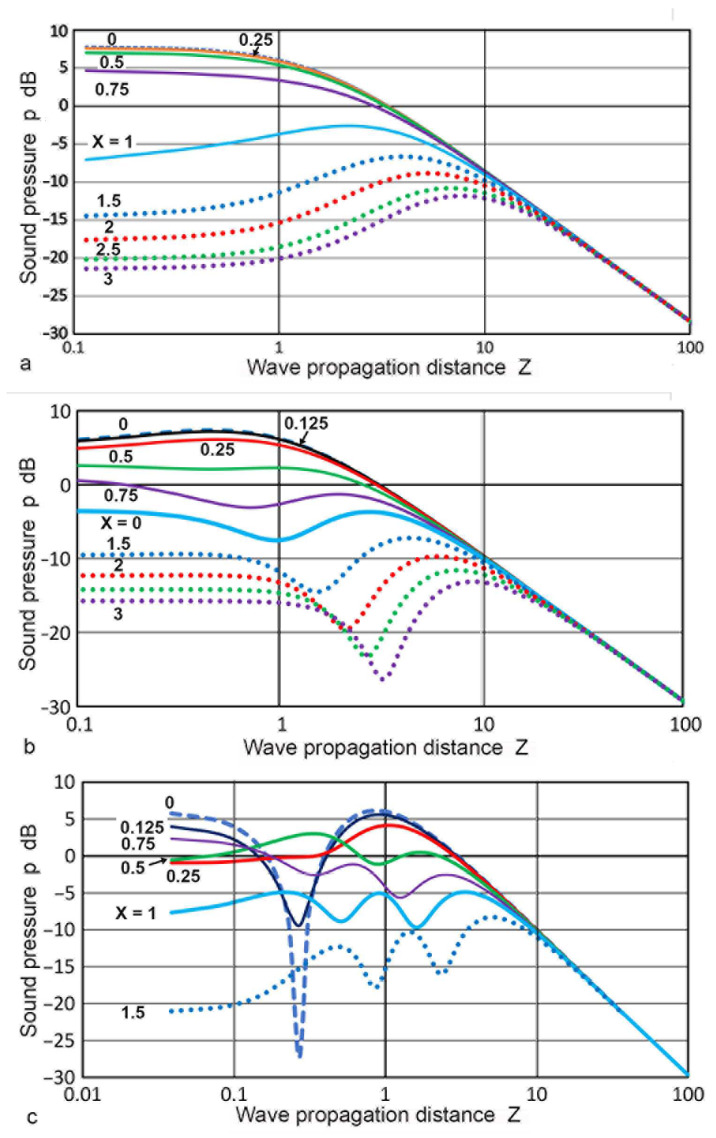

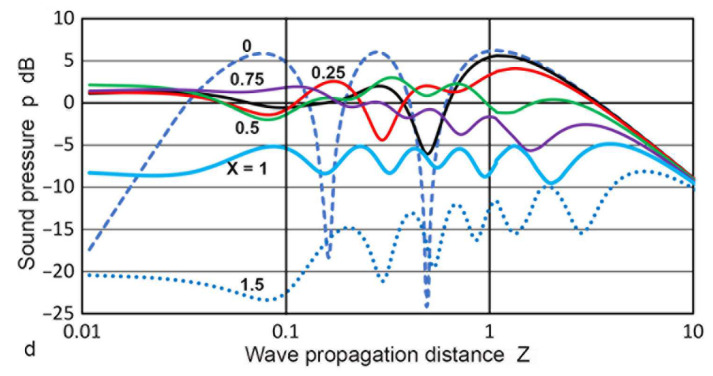
(**a**) Sound pressure, **p**(**X**,**Z**) vs. **Z** for various **X** values. The Zemanek model with a transmitter radius of 3.2 mm. **X** = 0: blue dash curve, 0.125: black (hidden), 0.25: red, 0.5: green, 0.75: purple, 1.0: light blue, 1.5: blue dot, 2.0: red dot, 2.5: green dot, 3.0: purple dot. (**b**) **p**(**X**,**Z**) vs. **Z** with an **a_ct_** of 5.0 mm. Color code for **X** same as in (**a**). (**c**) **p**(**X**,**Z**) vs. **Z** with an **a_T_** of 9.5 mm. Same color code for **X** except **X** ≥ 2 not included. (**d**) **p**(**X**,**Z**) vs. **Z** with **a_T_** of 19 mm. Same color code for **X** as in (**c**).

Four representative sets of calculated values of **p**(**X**,**Z**) against **Z** for **a_T_** values of 3.2, 6.4, 9.5, and 19 mm were plotted in Figure 7a–d, typically for **X** values of 0, 0.063, 0.125, 0.25, 0.5, 0.75, 1.0, 1.5, 2, 2.5, and 3. Depending on the **a_T_** value used, some low and high **X** values were not used. These figures correspond to Figure 3a, obtained using the Torikai equations. Results of the Zemanek calculations are dependent on the **a_T_/λ** ratio and a specific calculation is needed for each transmitter radius. All of the output was normalized at **X** = 0 and **Z** = 1, assigning **p** = 2.0 (or 6.0 dB). This step allows for a direct comparison with the output of the Torikai equations. Figure 7a shows the case of **a_T_** = 3.2 mm (with **a_T_/λ** = 0.54). The **p** values decreased smoothly with **Z** and merged to the inverse-**Z** slope at **Z** of 6 to 30. The top blue-dash curve is **X** = 0, or on-axis, while the bottom purple dotted curve is **X** = 3 or 9.6 mm from the center. The light-blue curve shows **p** values on the transmitter edge (**X** = 1) and the **p** value at **Z** = 1 is approximately 10 dB (a factor of 3.2) lower than **p** at the center. At 6.4 mm from the center (red-dotted curve, **X** = 2), the **p** value decreased another 12 dB at **Z** = 1. Differences in **p** between the center and outside points diminished with increasing **Z**. For smaller **a_T_** values, the trends were similar, but decreases at low **Z** for **X** ≥ 1 were absent and **p** values behaved like those for **X** < 1. Figure 7b shows the case of **a_T_** = 5.0 mm (with **a_T_/λ** = 0.85). The **p** values decreased smoothly with **Z** for **X** ≤ 0.5, while a dip started to appear in the **p–Z** curves for **X** ≥ 0.75. For **a_T_** = 6.4 mm (with **a_T_/λ** = 1.08), the trends were similar, but the dips became deeper and the low-**Z** decreases of **p** values for **X** ≤ 0.25 were larger, resulting in a peak at **Z** = 1. Figure 7c,d shows the cases of **a_T_** = 9.5 and 19 mm (with **a_T_/λ** = 1.7 and 2.6). The **p** values started to exhibit more complex behavior. For the on-axis curves in blue dash, two and three peaks developed. In Figure 7c, the top two curves (**X** = 0 and **X** = 0.125 in black) showed large dips of 33 and 15 dB at **Z** of 0.27. For the 19 mm radius, the corresponding dips in Figure 7d were at **Z** = 0.49 with depths of 30 and 12 dB. These dips represent the effects of the diffraction phenomena, as shown previously in Figure 3a. In both cases, the peak positions were close to **Z** = 1 for **X** ≤ 0.25. When **X** became larger, it shifted to low or high **Z** depending on **X**. This was also the case in Figure 7b.

**Figure 8:**
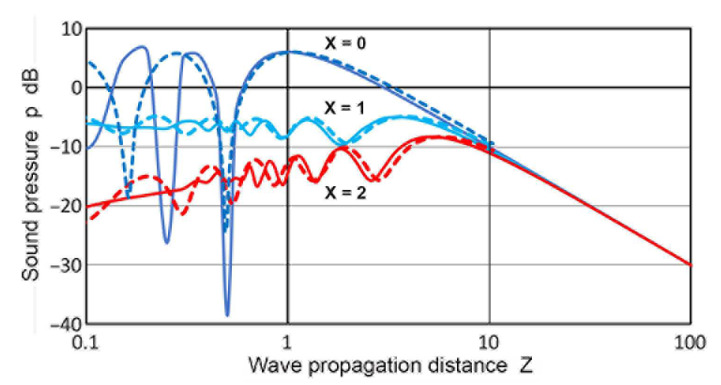
Comparison of the sound pressure, **p**(**X**,**Z**) vs. **Z** for **X** values of 0, 1, and 1.5 between the Torikai equations (solid curves, from Figure 3a) and the Zemanek model with a transmitter radius of 19 mm (dashed curves, from Figure 7d).

Figure 8 compares the variations in the **p** values at **X** = 0, 1, and 1.5 for the two calculation methods. Solid curves represent the Torikai equations and the dashed ones are from the Zemanek model using **a_T_** = 19 mm. General trends agreed between the two except for **X** = 0 at **Z** < 0.3. For on-axis or **X** = 0, the dip at **Z** = 0.5 was 12 dB deeper for the Torikai equations than the Zemanek model. In this region, the Torikai equations resulted in many dips at lower **Z**, although only three of them are shown. As shown in Figure 3a, these dips for the Torikai calculation vanished as **X** increased to 0.25. A similar trend was also found in Figure 7d for the Zemanek model. These low-**Z** dips produced limited effects on the integrated pressure plots (cf. Figure 4), which showed their effects only in two cases of the lowest **X′**s. Still, this can introduce sharp changes in the construction of DGS plots for small flaw sizes. The presence of these dips at or near the central axis is well-established [1,51], and the number of dips is related directly to the **a_T_/λ** values. This correlation was predicted for the Zemanek model [17] and was observed as noted earlier. No dip appeared for **a_T_/λ** smaller than unity, which was also shown in the original work [17]. In contrast, it is unclear how the Torikai equations became independent of the **a_T_/λ** values and implicitly assumed an infinite **a_T_/λ** because of the use of the cos(π/**z**) function in Equation (4). This may not preclude using them since Figure 8 showed reasonable agreement with the Zemanek model, but further study is needed.

**Figure 9:**
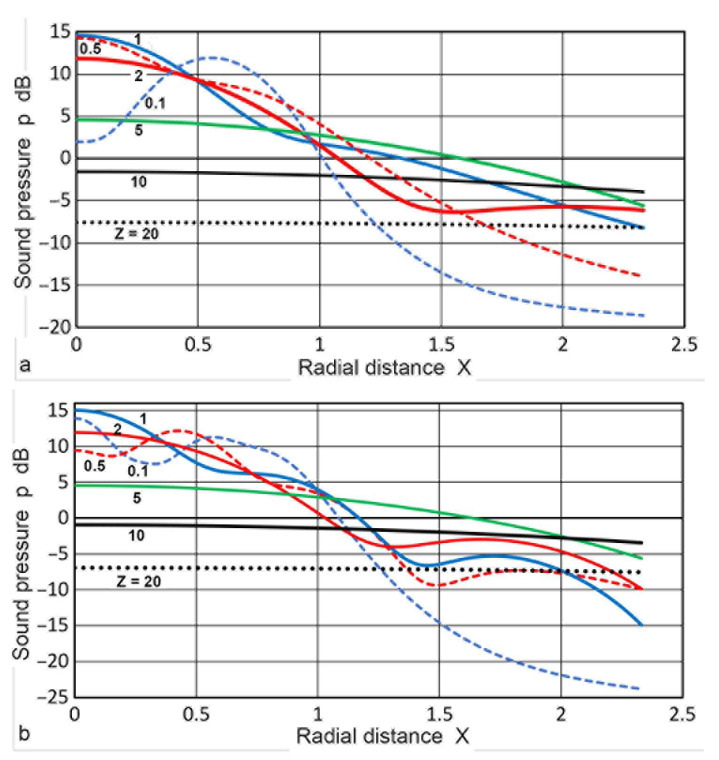
Sound pressure, **p**(**X**,**Z**) vs. normalized radius, **X**, at various values of **Z** for the Zemanek model. (**a**) **a_T_** = 6.4 mm. (**b**) 12.7 mm. **Z** = 0.1: blue dash, 0.5: red dash, 1.0: blue, 2.0: red, 5.0: green, 10: black, 20: black dot.

**Figure 10:**
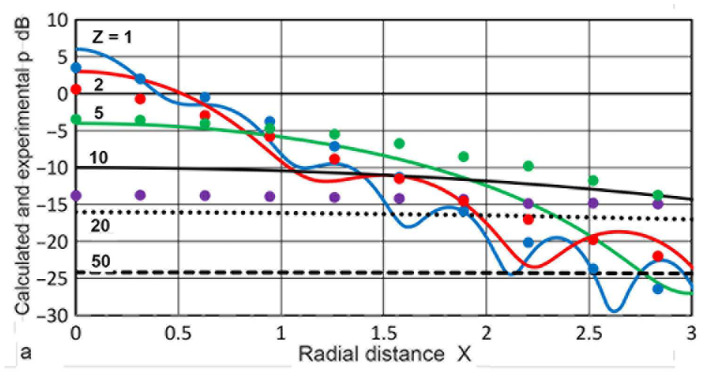

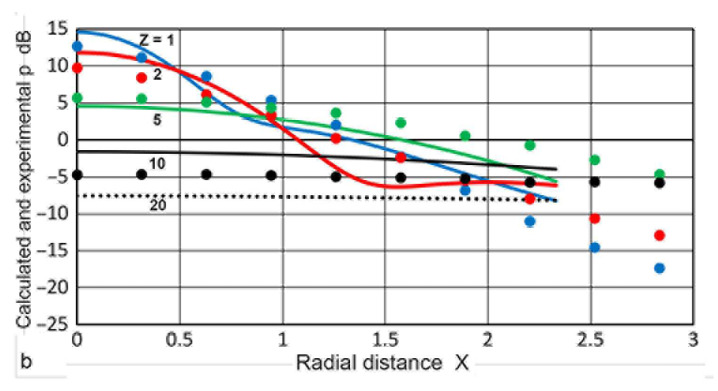
Experimental measurements of sound pressure, **p**(**X**,**Z**) vs. normalized radius, **X**, with **a_T_** = 6.35 mm at four values of **Z**. **Z** = 0.94 (blue dots), 1.89 (red), 3.75 (green)m and 16.10 (black). (**a**) Measured values were compared to the Torikai calculations at **Z** = 1, 2, 5, 10, 20, and 50 from Figure 4. (**b**) Comparison to the Zemanek model at **Z** = 1, 2, 5, 10, and 20 with the matching a_T_ of 6.4 mm (Figure 9a).

Changes in **p**(**X**,**Z**) calculated with the Zemanek model for **a_T_** = 6.4 and 12.7 mm were plotted in Figure 9a,b against **X** values of 0 to 2.3. The **Z** values were 0.1 to 20. The variations in the **p** values showed generally similar trends, as in the case of the Torikai equations (given in Figure 4), but less oscillations were evident for the low **Z** curves, except for **Z** = 0.1 in Figure 9a. For **Z** of 5 to 20, changes in the **p** values were comparable among the three. The calculated **p–X** curves were compared to the experimental results. Using a 1-MHz, 12.7 mm diameter transmitter (SFZ57A2439, Automation Industries, Chicago, IL, USA), a home-made pulser (–310 V, 0.05 µs risetime), four aluminum plates (6.40, 12.9, and 25.60 mm thick Al-6061 and 110.0 mm thick Al-7075), and a 1 mm diameter receiver (KRNBB, KRN Services, Richland, WA, USA), the values of **p**(**X**,**Z**) were determined. **Z** values were 0.94, 1.89, 3.75, and 16.10, respectively. Results of the peak-to-peak voltages of the first-arriving pulse (in dB) were plotted in Figure 10a,b against **X**. Experimental details of such tests are given in reference [52]. Attenuation correction of 1.5 dB was applied to the Al-7075 results using the attenuation data for this plate [33]. Their general trends agreed with the three calculated **p**(**X**,**Z**). The amplitude level for the 110 mm plate was normalized to correspond to the **p** value for **Z** = 16 at low **X** values. While minor differences in **Z** values were present, the variations in **p** values between **X** = 0 and **X** = 3 were comparable to the calculated results. While more extensive experimental studies are desirable, the observed changes in the sound field amplitude indicate that the present calculations offer a foundation for determining DGS diagrams with confidence.

**Figure 11:**
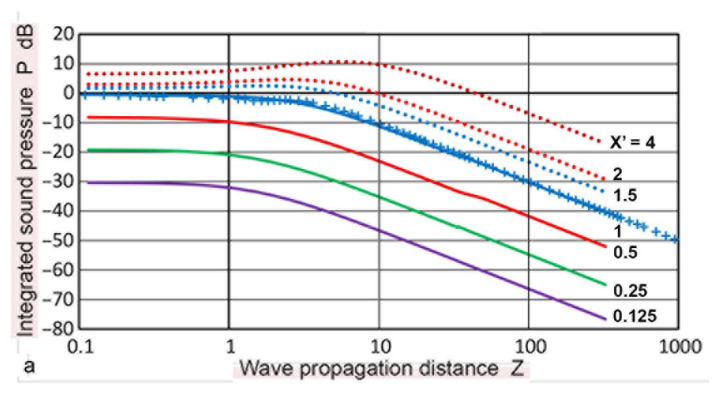

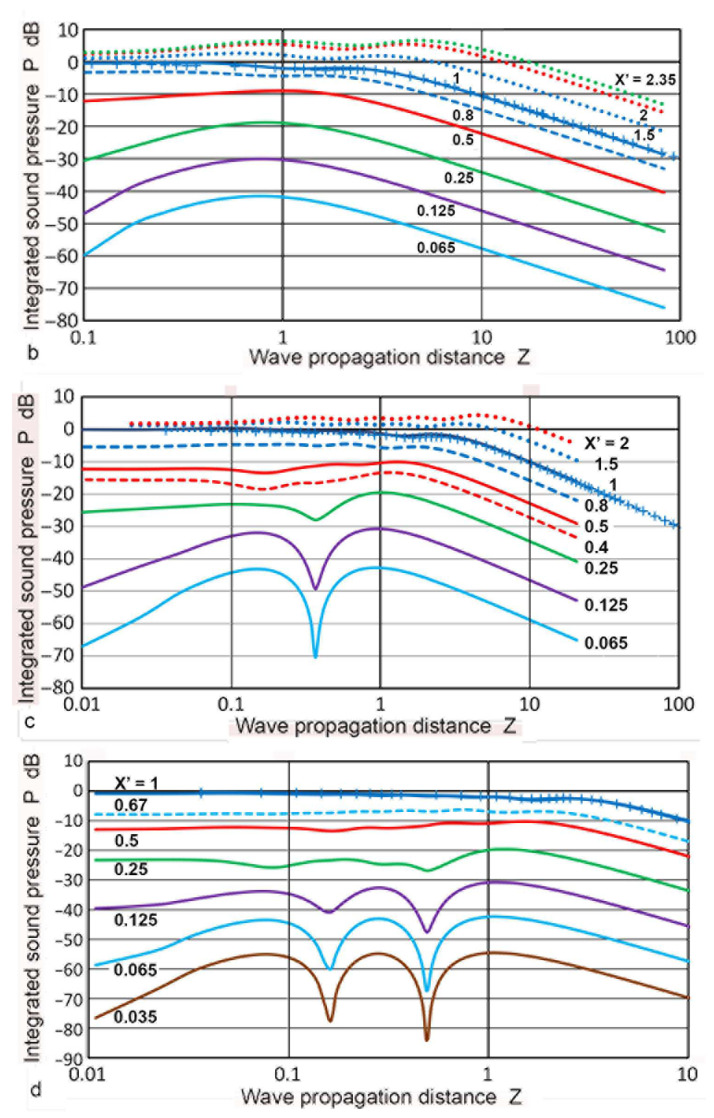
Integrated sound pressure, **P**(**X′**,**Z**) vs. **Z** using the Zemanek model. (**a**) **a_T_** = 3.2 mm. **X′** = 0.125: purple, 0.25: green, 0.5: red, 1.0: blue, 1.5: blue dot, 2: red dot, 4: dark red dot. **D**(**Z**) vs. **Z** in blue **+**. (**b**) **a_T_** = 6.4 mm. As in (**a**), plus **X′** = 0.065: light blue, 0.8: blue dash, 2.35: green dot. (**c**) **a_T_** = 12.7 mm. As in (**b**), plus **X′** = 0.4: red dash. (**d**) **a_T_** = 19 mm. As in (**b**), plus **X′** = 0.035: brown, 0.67: light blue dash.

Using the **p**(**X**,**Z**) calculations described above, sectional integration was again applied to obtain **P**(**X′**,**Z**) values for all 11 cases calculated. Of these, four cases of **a_T_** = 3.2, 6.4, 12.7, and 19 mm were chosen and their **P** vs. **Z** plots are given in Figure 11a–d. All of the calculation results converted to dB scale were normalized by subtracting 151.6 dB. This normalization was carried out to match **P**(1,**Z**) with the **D** values from the TRvB equation. Since the **a_T_** value differs from one case to another, the **Z** values changed accordingly, since the Zemanek calculations were conducted using **Z′** = **z**/**λ**. In Figure 11a–d, the **D** values were superimposed with **+** symbols on the curve for **P**(1,**Z**), plotted in blue.

### 2.4. Comparison of Two Calculation Results

**Figure 12:**
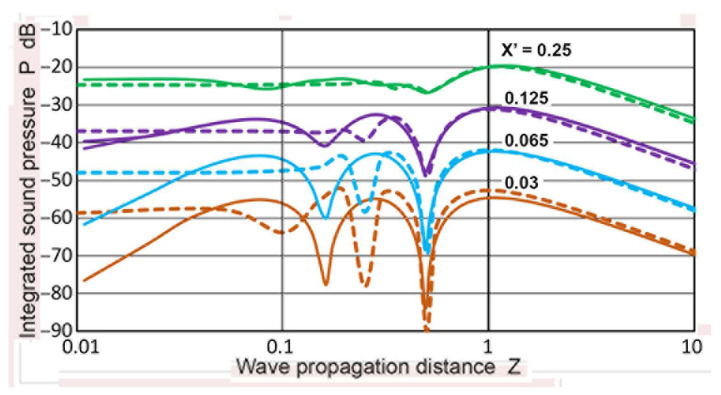
Comparison of the integrated sound pressure, **P**(**X′**,**Z**) vs. **Z** curves from the Torikai (dash curves) and Zemanek model (**a_T_** = 19 mm, solid curves) calculations (Figure 5 and Figure 11d). Four **X′** values were used—0.03: brown, 0.065: light blue, 0.125: purple, 0.25: green.

Figure 11a shows **P**(**X′**,**Z**) vs. **Z** for **a_T_** = 3.2 mm. The bottom curve is for **X′** = 0.125, followed by plots for **X′** = 0.25 to 4.0. The general trend resembled Figure 5 for the Torikai calculations. All of the **P–Z** curves at low **Z**s were nearly flat for **X′** ≤ 1. It was noted that the **P** values for **X′** > 1 decreased slowly at **Z** < 10, unlike the Torikai results. In the far-field region, the **P** values for a constant **Z** increased in a 12 dB step for the doubling of **X′** values, indicative of the **X′**^2^-dependence. Figure 11b shows **P**(**X′**,**Z**) vs. **Z** for **a_T_** = 6.4 mm with **X′** values from 0.063 to 2.35. The main features were nearly identical, but **P** values for **X′** > 1 decreased at lower **Z** values, similar to the trend observed in Figure 4. Another change was a decreasing trend for **Z** < 1 for lower **X′** curves, resulting in maxima near **Z** = 1. The next figure shows the case of **a_T_** = 12.7 mm. The range of **X′** was from 0.063 to 2 including 0.4 and 0.8. Again, 12-dB differences in **P** in the far-field region were mostly present for a doubling of **X′**, but a significant change was observed for lower **X′**. Variations of **P** with **Z** for **X′** ≤ 0.5 now showed a dip in the low **Z** region, as observed earlier in Figure 5. The positions of the dips were at **Z** = 0.37. The emergence of similar dips in the **P–Z** curves was also observed for the case of **a_T_** = 9.5 mm, and their positions were at **Z** = 0.26. Below the dips, the **P–Z** curves for **X′** ≤ 0.5 increased with **Z**, followed by a downward trend for **Z** > 1. Figure 11d shows **P**(**X′**,**Z**) vs. **Z** for **a_T_** = 19 mm, showing the same general trend as in Figure 11c. The range of **X′** was from 0.03 to 1 including 0.67. Similarly to Figure 5, the **P–Z** curves developed two prominent dips for the three lowest curves. The dip positions at **Z** = 0.5 were identical to the dip positions in Figure 5. These two sets of **P–Z** curves were plotted together in Figure 12. For **X′** = 0.25, the curves matched well for **Z** > 0.3. At lower **X′**, the general trends agreed for **Z** > 0.3 as the second dip positions shifted for lower **Z** in the Zemanek model. The **P–Z** curves for the Zemanek model also decreased below the lowest peak.

**Figure 13:**
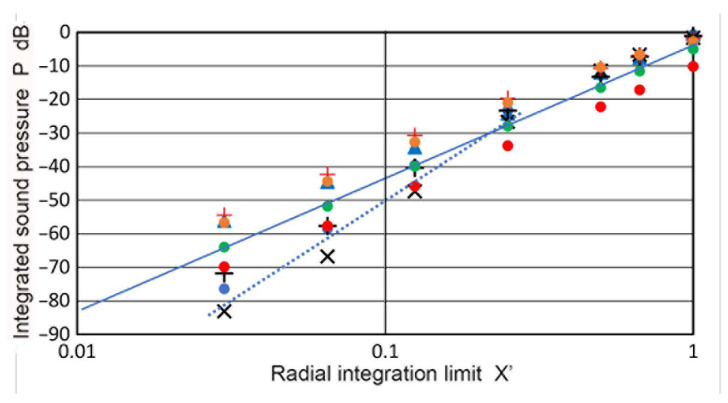
Integrated sound pressure, **P**(**X′**,**Z**) vs. radial integration limit, **X′** for different **Z** values with **a_T_** = 19 mm. **Z** = 0.01: blue dot, 0.05: blue triangle, 0.15: black **+**, 0.5: black X, 1: red **+**. 2: orange dot, 5: green dot, 10: red dot. Blue line through green dots represents **X′**^2^ or the slope = 2. Blue dotted line is for **X′^3^** or the slope = 3.

As in the case of the Torikai calculation, the **P**(**X′**,**Z**) values in all of the Zemanek calculations showed that the **p** values in the far-field region were proportional to the receiver area or **a_R_**^2^. When the transmitter size was small (e.g., **a_T_** = 6.4 mm), the same **X′**^2^-dependence was also found in the near-field region. For larger **a_T_** values, differences developed, as shown in Figure 13. This was the case of **a_T_** = 19 mm. For a **Z** of 1 to 10, again, **X′**^2^-dependence appeared. However, the Zemanek calculations in the near-field region mostly had a higher slope of close to three, or **X′**^3^ dependence. Specifically, the data for **Z** of 0.01, 0.15, and 0.5 exhibited a slope of 3 (shown by a dotted blue line). An exception was for **Z** = 0.05, with a slope of two. Some parts of the near-field region near the dips were unsuited for slope-fitting, thus, it was difficult to generalize **X′** dependencies of the integrated sound pressure in the near-field region.

From the above comparison of the two computational approaches, differences were most prominent between the two calculations in the near-field region. Figure 11a–d clearly show that the effects of the transmitter size must be properly considered. This arose from the independence of **P**(**X′**,**Z**) on **a_T_/λ** for the Torikai calculation. While the exact reason for the **a_T_/λ-**independence has not been clarified, it is likely to come from the use of a geometrical approximation in the derivation of the Lommel formulas. This has been recognized since the early stage of sound field studies (e.g., [12,13,14,15,16,17,18,19]). In fact, parts of the near-field region had to be excluded, as shown in Figure 5. Thus, it is preferrable to rely on the Zemanek model and other simulation tools, using the Lommel formulas only for reference.

In this section, the sound field of a circular transmitter was obtained using the two methods of Torikai and Zemanek, and the results were described and compared. In the far-field, both results were consistent in terms of **p**(**X**,**Z**) and **P**(**X′**,**Z**) and mostly matched well. In the near-field, parts of the Torikai results were outside the Lommel limit and the Zemanek results varied with the **a_T_/λ** ratios, as predicted. When the integrated values, **P**(**X′**,Z), were examined, the differences were less for larger **a_T_/λ** ratios, even in the region excluded by the Lommel limit. However, the **a_T_/λ**-independence of the Torikai equations is a serious flaw in dealing with the near-field behavior, and Zemanek calculations will be used with more weight in the following sections.

## 3. DGS Diagrams

### 3.1. Forward and Backward Diffraction

In order to construct a DGS diagram, it is necessary to use a series of transmitter-to-reflector paths and reflector-to-receiver paths for various transmitter–reflector pairs of different sizes. These are forward and backward paths corresponding to Figure 1b,c, when a reflector is smaller than the transmitter. For a larger reflector, the order is reversed to Figure 1c,b. In the backward path, the reflector acts as a transmitter and the initial transmitter is used as a receiver. It is necessary to define parameters for forward and backward paths. These are tabulated in Table 1.

For the forward path, **S** = **a_R_/a_T_** and the near-field distance, **N**, were as defined previously. For the backward path, the reflector acts as a transmitter with the size of **a_T′_** = **a_R_**, having the near-field distance of **N_B_** = **N S^2^**. The original transmitter becomes a receiver with its size being **a_R′_** = **a_T_**. Thus, **N_B_** becomes very small for a small reflector.

**Figure 14:**
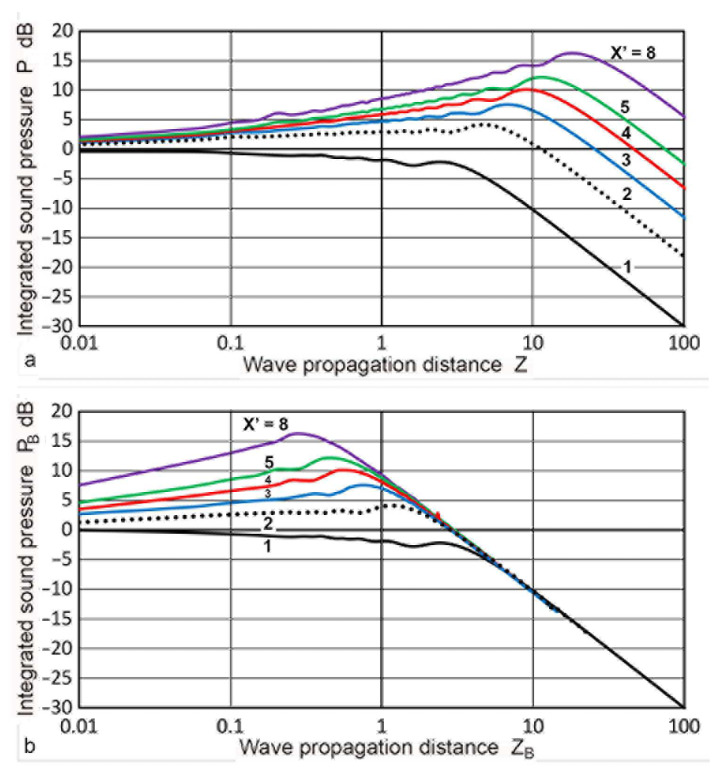
Integrated sound pressure, **P**(**X′**,**Z**) vs. **Z** using the Torikai equations. (**a**) Forward path case. (**b**) Backward path case. **X′** = 1: black curve, 2: black dotted, 3: blue, 4: red, 5: green, 8: purple.

Consider first the forward path case of the Torikai equations. The **P**(**X′**,**Z**) values for **X′** of 0.065 to 3 were previously plotted against **Z** in Figure 5. An expanded plot for 1 ≤ **X′** ≤ 8 is given in Figure 14a. For **X′** = 2, the **P** values showed a maximum (4.25 dB) at **Z** = 5. At a larger **X′**, the peak position shifted to a higher **Z**; for **X′** = 8, the peak reached 16.2 dB at **Z** = 18. The peak value of **P**, **P_m_**, is proportional to **X′** and is given by **P_m_** = 0.812 **X′** (R^2^ = 0.9999).

Under the backward path condition, a large reduction in **N_B_** occurred as **X′** increased. For example, as **X′** went from 1 to 8, **S_B_** = 1/8, and **N_B_** = 0.0156**N**, it shifted the purple curve in Figure 14a by a factor of 64 (or 1.81 decade) to the left. After applying the shifts, the **P_B_**(**X′**,**Z_B_**) vs. **Z_B_** curves became aligned with the far-field portion of the **P**(1,**Z_B_**) curve, as shown in Figure 14b. Here, **P_B_**(1,**Z_B_**) is plotted in black and **P_B_**(8,**Z_B_**) in purple, while the **P_m_** values remained unchanged. Before this figure was plotted, the unit of **Z_B_** was converted for each curve to that of **Z**, defined in the unit of **N** for the transmitter size, **a_T_**, making this scale identical to that used in Figure 5 for the forward path. This figure shows that the **P_B_** vs. **Z_B_** relation is unaffected by **X′** in the far-field, but is strongly affected by both **X′** and **Z** in the near-field. In order to obtain Figure 14b, an extra step was needed since **N_B_** is different for each curve since **a_T′_** is different. After tabulating **P_B_** and **N_B_** for an **X′** value, the **P_B_** value was read by interpolation for each **Z** value (in **N**) utilized for the forward path. This was needed for the present method of computation of the Torikai equations, but the results allowed for arithmetic operations of **P** and **P_B_** values at identical wave propagation distances. Alternately, an entire Torikai computation could be repeated for each **X′** value used. While this is feasible, it was impractical for the Excel-based method used here.

### 3.2. Echo Amplitude Determination

When a reflected echo returns to the transmitter, the integrated sound amplitude of the echo, or echo amplitude, **G**, is given by the sum of the forward and backward amplitude values (both in dB scale) as

**Figure 15:**
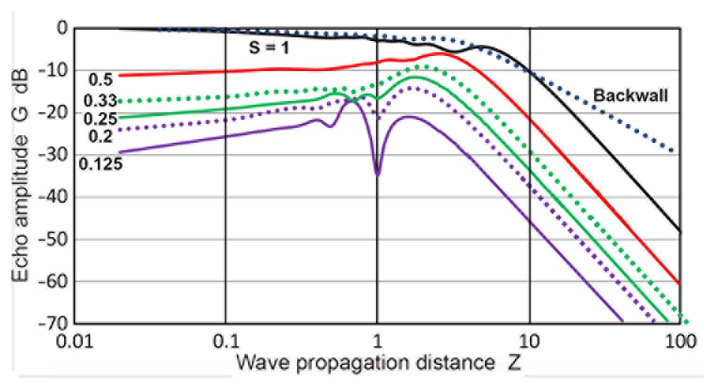
DGS diagram using the Torikai equations. **S** values are 0.125 (purple curve), 0.2 (purple dotted), 0.25 (green), 0.33 (green dotted), 0.5 (red), and 1 (black). **D** vs. **Z** is plotted in the black dotted curve, taken as the backwall reflection.

**G**(**S**,2**Z**) = **G**(**X′**,2**Z**) = **P**(**X′**,**Z**) + **P_B_**(**X′**,**Z**), (6)

where the total wave propagation distance is given by 2**Z** and both **P**(**X′**,**Z**) and **P_B_**(**X′**,**Z**) are given in dB scale. Note that the summation must be taken at the same **z** (or **Z′**) for the two paths. In the Torikai calculation procedures described in the previous sections, this condition was not fulfilled automatically and repeated interpolation procedures had to be used to obtain proper **P_B_** values. Values of **G**(**S**,2**Z**) resulting from the summation are plotted in Figure 15. Since the Torikai formulas are independent of **a_T_/λ**, this is the unique representation of a DGS diagram from the Torikai calculation. In this diagram, **D** values were plotted as the black dotted curve. This curve was treated to represent the backwall reflection from the original Krautkramer construction [36], showing the 1/**Z** dependence for **Z** > 10. The next curve in black shows the case of **S** = 1, given by

**G**(1,2**Z**) = 2**D**(**Z**) = 2**P**(1,**Z**), (7)

showing 1/**Z**^2^ dependence for **Z** > 10. Again, this was used in Krautkramer [36]. Five additional curves were obtained from the Torikai formulas in this work for **S** = 1, 0.5, 0.33, 0.25, 0.2, and 0.125 (in black, red, green dot, green, purple dot, and purple curves). All of them showed a gradual rise from low **Z**s to a maximum (or maxima) and the 1/**Z**^2^ dependence for a higher **Z** above 3 to 8. For lower **S** values below 0.25, dips were observed at a **Z** of 0.5 to 1. These dips were previously observed in **P**(**X′**,**Z**) curves (Figure 5). In the far-field region, **G** values were proportional to **S**^2^ for a given **Z**. This was verified at **Z** = 10 and indicated that the average echo amplitude depends on the reflector area, validating the postulate used in the original DGS construction [36].

**Figure 16:**
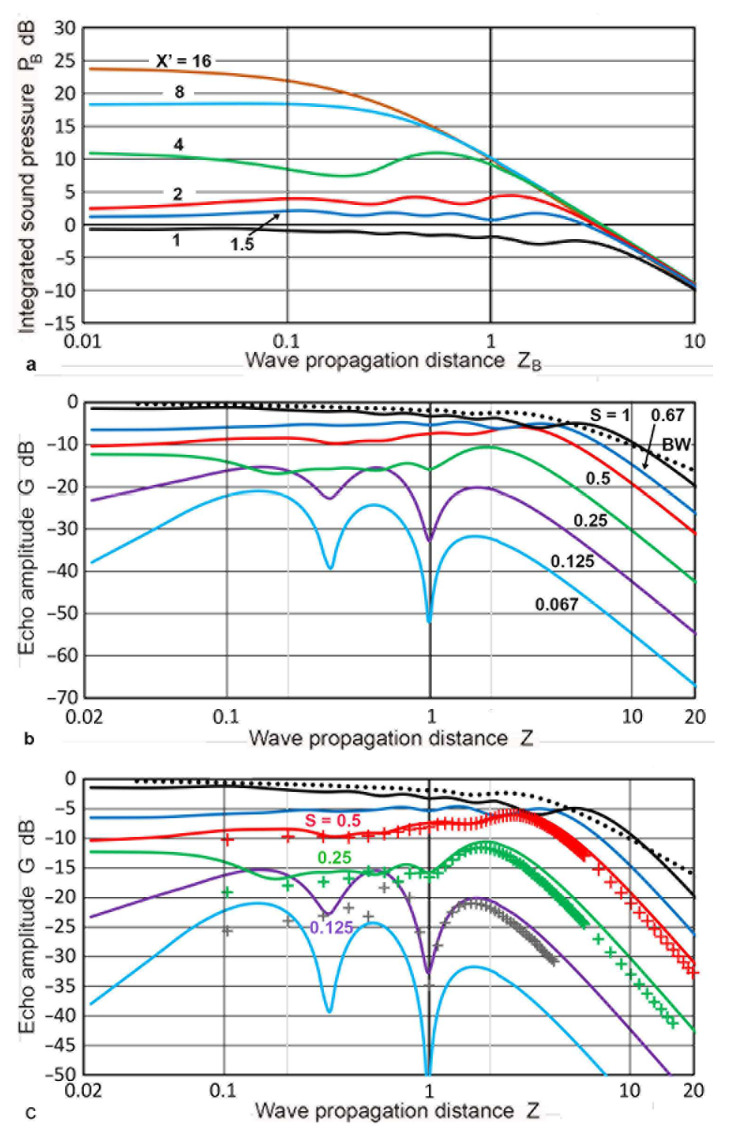
(**a**) Integrated sound pressure, **P**(**X′**,**Z_B_**) vs. **Z_B_** using the Zemanek model with **a_T_** = 19 mm. Converted to backward path. **X′** = 1: black curve, 1.5: blue, 2: red, 4: green, 8: light blue, 16: brown. (**b**) DGS diagram using the Zemanek model with an **a_T_** = 19 mm. **S** = 0.063: light blue curve, 0.125: purple, 0.25, green, 0.5: red, 0.67: blue, 1: black. **D** vs. **Z** in the black dotted curve. (**c**) The Zemanek DGS diagram with the Torikai DGS data co-plotted for **S** = 0.125, 0.25, and 0.5 using black, green, and red **+** symbols.

Calculations of **P**(**X′**,**Z**) based on the Zemanek model, were also used in constructing the DGS diagrams. For this approach, calculations with **a_R_** values of 6.4 mm or larger were used for the forward path. For the backward path, smaller **a_R_** values were of interest due to the presence of multiple dips in **G** values. All of the **P**(**X′**,**Z**) calculations were made using **Z′** = **z/λ** as the distance parameter, and switching between the forward and backward paths required no additional computational step (i.e., no need for interpolation). Results for the case of **a_T_** of 19 mm are presented first. The **P**(**X′**,**Z**) vs. **Z** plots are given in Figure 11d in the previous section. Plots of **P_B_**(**X′**,**Z_B_**) are given against **Z_B_** in Figure 16a. Since each of the curves was from a different set, the **P_B_** vs. **Z_B_** plotting of Figure 16a had to plot each curve separately. The unit of **Z_B_** was converted to **Z**, which used **N** for **a_T_** = 19 mm, or 54.31 mm. This was identical to the **Z** used for the forward path. The original data came from six sets of MATLAB calculations that used an **X** step of 0.4 mm for an **a_T_** of 1.2 to 19 mm. By combining **P_B_**(**X′,Z**) with **P**(**X′**,**Z**), the **G**(**X′**,2**Z**) values were obtained and plotted in Figure 16b, producing a DGS diagram with the Zemanek model for the 19 mm radius transmitter/receiver. The general trend of this DGS diagram matched well to that of Figure 15, which was calculated using the Torikai formulas. The main dips were at **Z** = 1, a gradual decrease in **G** with a **Z** decrease and **Z**^2^ dependence in the far-field region. Three series of data points from the Torikai DGS for **S** = 0.125, 0.25, and 0.5 were added to Figure 16b with **+** symbols using the same color schemes in Figure 16c. For **S** = 0.125, the positions of the dip at **Z** = 1 agreed, although the two curves diverged below a **Z** of 0.7. Data for two higher **S** values followed each other well within 3 dB. While these two DGS diagrams exhibited similar trends, it was noted that **a_T_/λ** = 19/5.9 = 3.22 for this Zemanek DGS, while the **a_T_/λ** was expected to be much larger for the Torikai DGS because of many zero-crossings of **p**(0,**Z**) for a **Z** of less than 0.1.

**Figure 17:**
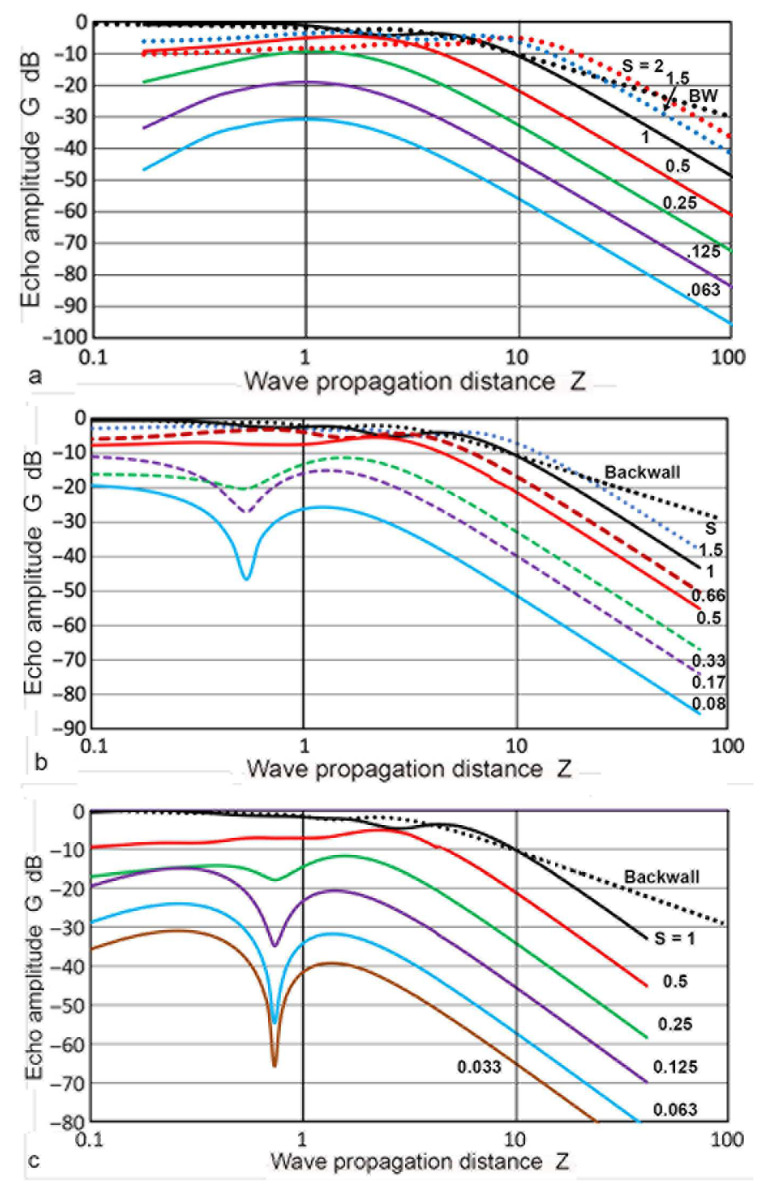

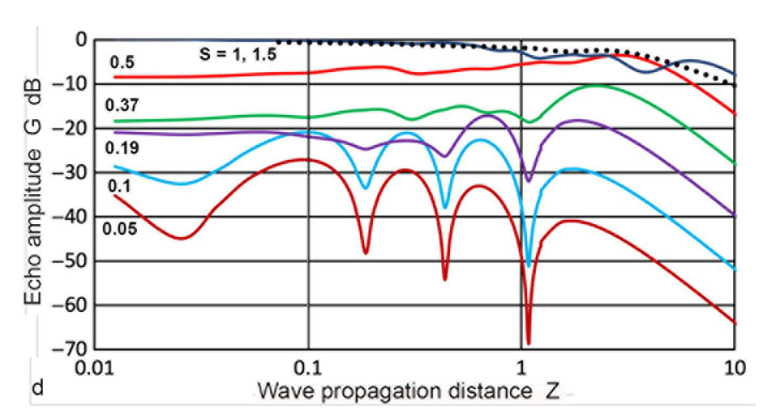
The Zemanek DGS diagrams for different transmitter sizes. (**a**) **a_T_** = 6.35 mm. **S** = 0.063: light blue curve, 0.125: purple, 0.25: green, 0.5: red, 1: black, 1.5: blue dotted, 2: red dotted. **D** vs. **Z** in the black dotted curve. (**b**) **a_T_** = 9.5 mm. **S** = 0.08: light blue curve, 0.17: purple dash, 0.33: green dash, 0.5: red, 0.66: red dash, 1: black, 1.33: blue dotted. (**c**) **a_T_** = 19 mm. **S** = 0.04: brown, the rest same as in a. (**d**) **a_T_** = 25.4 mm. **S** = 0.05: brown, 0.1: light blue, 0.19: purple, 0.37: green, 0.5: red, 1: blue. 1.5: black dot.

A further four Zemanek DGS diagrams are shown in Figure 17. These were for an **a_T_** of 6.4, 9.5, 12.7, and 25.4 mm (**a_T_/λ** = 1.08, 1.61, 2.15, and 4.3). For an **S** higher than 0.5, the curves were nearly identical. For the smallest **a_T_** value of 6.4 mm (Figure 17a), all of the curves had a broad peak with no dip at all. As the **a_T_** value increased, the lower **S** curves started to develop a dip each. Two curves at **S** > 1 were included. Both showed a peak and a decreasing **G** at lower **Z**, becoming lower than the unity **S** curve. In Figure 17b, the single dip was at **Z** = 0.54, while Figure 17d showed three dips for the largest **a_T_**. The depth of the dips also increased. In addition, the **Z** position of the main dips shifted to a larger **Z**, reaching **Z** = 0.74 for an **a_T_** = 12.7 mm. This trend continued in the cases of **a_T_** = 19 and 25.4 mm. For the two largest **a_T_** (19 and 25.4 mm), the positional change was small, from 1.0 to 1.09, suggesting less effects for larger transducers.

**Figure 18:**
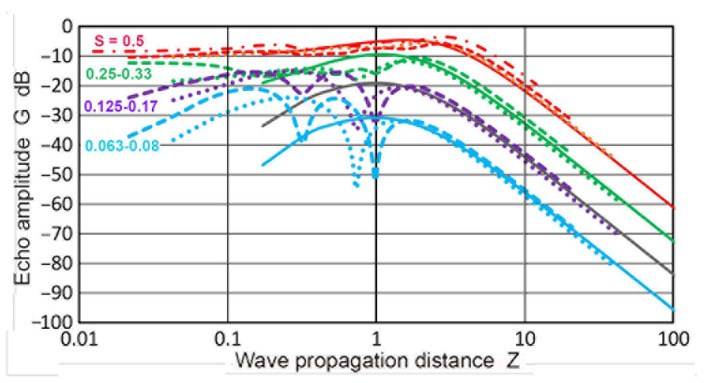
Comparison of the Zemanek DGS diagrams for different transmitter sizes. **a_T_** = 6.35 mm. Solid curves, a_T_ = 9.5 mm. Dotted, **a_T_** = 19 mm. Dash, **a_T_** = 25.4 mm. Dash-dotted. **S** = 0.063–0.08: light blue, 0.125–0.17: purple, 0.25–0.33: green, 0.5: red.

The effects of the **a_T_/λ** variation on the shape of the DGS curves were examined in Figure 18 by co-plotting the **G–Z** curves together for an **a_T_** of 6.4 to 25.4 mm. The top group had four red curves for **S** = 0.5. These varied smoothly and the differences were small, not exceeding 5 dB. The second group consisted of three green curves for **S** = 0.25 (omitting an **a_T_** of 25.4 mm, which did not have this **S** value). For the two larger **a_T_**, dips started to develop and their curves were lower near **Z** = 1. While the curves matched those of **Z** > 1.8, they started to diverge below a **Z** of 0.3. The purple curves of **S** = 0.125 were close together down to **Z** = 1.3. Dips were present for the larger **a_T_** and the trends differed from the 6.35 mm transducer below a **Z** of 1.3. All three did move downward at low **Z**. These features also appeared for **S** = 0.063 (light blue curves). Three peaks developed for the largest **a_T_**, whereas only one peak at **Z** = 1 was retained for the smallest **a_T_** case. This comparison indicates that the primary effects of **a_T_/λ** variation were noticeable in the near-field region.

The Zemanek DGS diagrams were clearly dependent on **a_T_/λ**. This effect was absent in the Torikai DGS diagram, but it matched that of the Zemanek DGS at **a_T_/λ** of 3.22. Since the source of **a_T_/λ** independence is unknown for the Lommel approach, it appears prudent to avoid the Torikai equations. Instead, the Zemanek model or its improved versions provide a firm foundation for DGS determination based on the two-way diffraction approach.

## 4. Discussion

The available DGS diagrams were examined next using the sound fields and DGS diagrams produced in this study. In the preceding section, the DGS diagrams were determined using sound field calculations based on the two different approaches of Torikai and Zemanek. Integrated sound fields were obtained for both the forward and backward wave propagation paths. These were combined by the two-way diffraction methodology. The results were used next to evaluate the published DGS curves in order to assess the methods of their construction. As noted earlier, the methods used previously have not been disclosed, apparently being treated as proprietary information.

**Figure 19:**
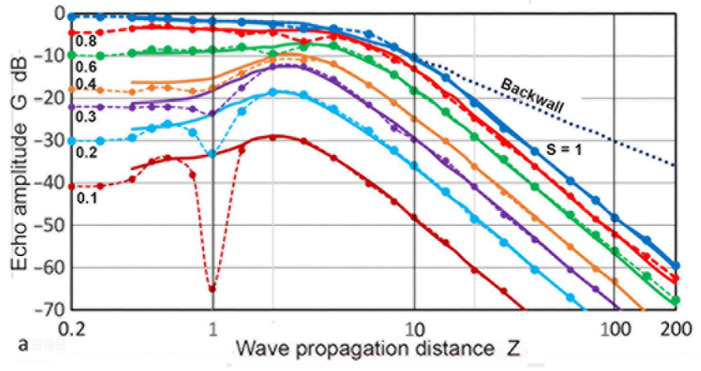

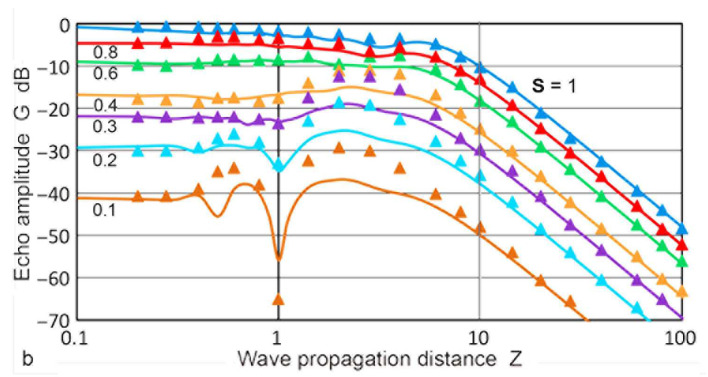
Comparison of the DGS diagrams. (**a**) Data points of the Mundry DGS curves (connected by dash curves) and the general DGS curves from ISO 16811 (solid curves). **S** = 0.1: brown, 0.2: light blue, 0.3: purple, 0.4: orange, 0.6: green, 0.8: red, 1: blue. Backwall: black dotted. (**b**) Data points of the Mundry DGS curves in triangular symbols and quasi-DGS curves based on the Torikai calculation (solid curves). Same color codes as in (**a**).

Mundry and Wüstenberg [38] published a DGS diagram based on the calculated sound fields. Their calculation was limited to the region of **Z** ≤ 5, and the results from Krautkramer [36,37] were used for the far-field region. Their DGS data points were selectively read and replotted in Figure 19 with circle symbols and connected with dash curves, with color coding for the **S** values. Here, the total propagation distance was used as the **Z** and **S** values were between 0.1 and 1. At an **S** below 0.2, dips in **G** were observed and the depth reached 36 dB from the nearest maximum. The general DGS curves, read from the general DGS diagram in ISO16811, were also plotted in Figure 19 [49]. These were in solid curves of the same colors as the Mundry curves with matching **S** values. When the dips were ignored, the two sets of DGS curves matched nearly perfectly. This strongly indicated that the origin of the general DGS diagram in ISO 16811 was originally published by Mundry and Wüstenberg [38]. The main differences were the smoothing of the dips in the Mundry curves. This data alteration process was without justification or disclosure, making ISO 16811 unfit to be an international standard. The absence of dips in the near-field region was observed in the Zemanek model calculations, as shown in Figure 17a. This was for the case of **a_T_/λ** = 1.08. However, the dips started to appear when the **a_T_/λ** increased to 1.68 (Figure 17b). Therefore, it is necessary for the authors and reviewers of ISO 16811 to demonstrate that the absence of the dips originated from the appropriate theoretical calculations or experimental evidence. Indeed, Krautkramer [36,37] relied on experiments in the near-field region. No such verification was included in ISO 16811 [49].

Figure 19b again shows the Mundry DGS data points (in triangular symbols). Solid curves were obtained as quasi-DGS curves using the Torikai calculations. These curves combined the **P**(**X′**,**Z**) vs. **Z** curves (cf. Figure 5) for the forward path and the **D**(1,**Z**) values from the TRvB equation for the backward path. This combination followed the method described by Krautkramer [1], which ignored the need to properly account for the backward diffraction. This procedure led to **S**^2^ dependence at low **Z** and 1/**Z**^2^ dependence in the far-field region. An excellent agreement was observed between the Mundry DGS and quasi-DGS curves based on the Torikai calculation excluding the region near the dips up to **Z** of 5. Good matches at low **Z** range are evidence that the Mundry curves were obtained using the approximate method described in Krautkramer [1]. Note that the **S**^2^ dependence of the integrated sound pressure, **P**, at low **Z**, was present in the sound field calculations (cf. Figure 6). Thus, this dependence proved that the backward path was independent of **S**, also implying the use of the **D–Z** function. This is why a good agreement resulted in Figure 19b. This use of the TRvB equation (applicable only to the case of equal-sized transmitter–receiver) for the backward path sound fields (represented by Figure 1c) is without foundation. Such a procedure cannot be justified for the valid construction of DGS diagrams. Thus, the Mundry DGS diagram was constructed by an invalid procedure and must be treated as an approximation. Since it was apparently used as the basis of the general DGS diagram in ISO 16811, this ISO standard needs to be treated as an invalid document.

**Figure 20:**
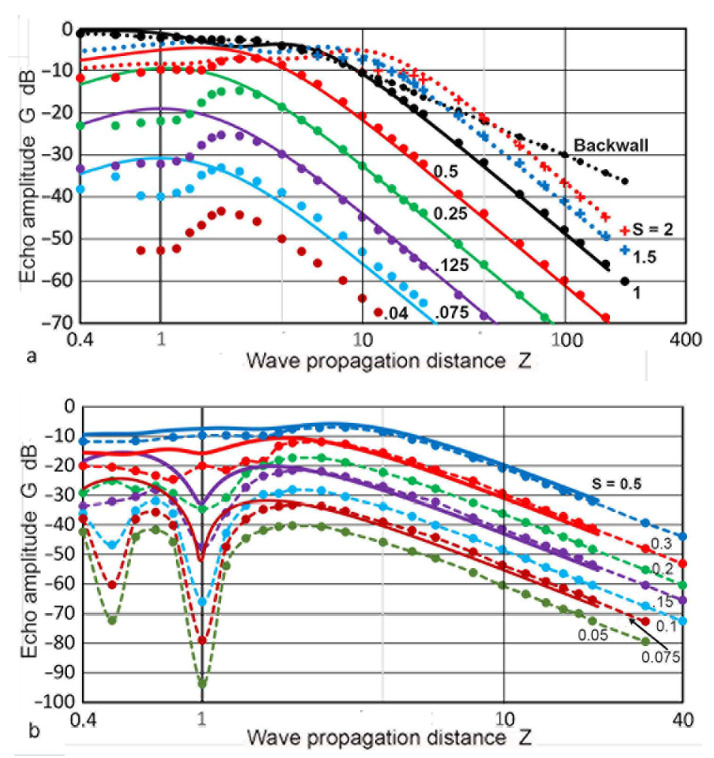

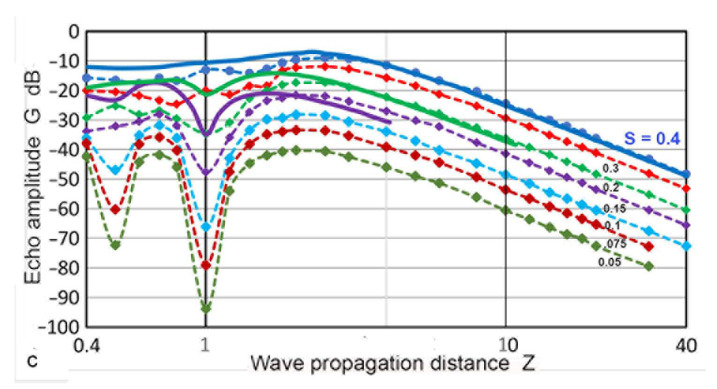
Comparison of the DGS diagrams. (**a**) Data points of the Kimura DGS curves (in dots for **S** ≤ 1 and **+** symbols for **S** > 1) with maximized **G** values. The corresponding DGS curves for an **a_T_** of 12.7 mm are given by the solid curves. **S** = 0.04: brown, 0.065/0.075: light blue, 0.125: purple, 0.25: green, 0.5: red, 1: black, 1.5: blue, 2: red dot. **D** vs. **Z** is shown by the black dots. (**b**) Data points of the Kimura DGS curves without **G** maximizing in dots; S = 0.05, 0.075, 0.1, 0.15, 0.2, 0.3, 0.5 (from low to high). Four Zemanek DGS curves for an **a_T_** of 38 mm with **S** = 0.065, 0.125, 0.25, and 0.5 with solid curves (brown, purple, red, and blue, respectively). (**c**) Kimura DGS data as in (**b**), but the top **S** of 0.4 was used. Torikai DGS curves are given for **S** = 0.125, 0.2, and 0.4 (purple, green, blue, respectively).

Kimura [41] published DGS diagrams based on the Torikai formulas. He also disclosed no details regarding the procedures used. However, his DGS diagrams included data for an **S** larger than unity, as shown in Figure 20a. This indicates that the second Torikai formula (Equation (3)) was utilized for backward-path diffraction. As observed in the Mundry DGS diagram (Figure 19a), the **G–Z** curves for an **S** below 0.5 showed a peak between **Z** of 2 and 4, but had no sharp dips. Kimura noted that this dataset was obtained by maximizing the **G** values by adjusting the position of the reflector areas. However, the adjusted areas were not described, making the low **Z** portion of this DGS diagram undefined. For comparison, DGS curves using the Zemanek model (with **a_T_** of 6.4 mm) were also plotted for **S** = 2 to 0.065 in Figure 20a. A good match was found for **Z** >3, but deviated from each other for lower **Z**. For the **S** of 1.5 and 2, the agreement was also good, except at **Z** <20 for **S** = 2. Kimura [41] also provided another set of **G–Z** curves with dips for **S** below 0.2, which were described as being due to on-axis reflectors. This transducer-reflector geometry is the normal one, as shown in Figure 1. The observed dips for low **S** curves occurred at **Z** = 1, as shown in Figure 20b. This dip position is consistent with those in the present calculations with the Zemanek model (with **a_T_** of 19 mm, Figure 16b) and with the Torikai formulas (Figure 15). Low **S** curves of these two DGS diagrams were inserted (shown in solid curves) in Figure 20b,c, respectively. However, the depths of the dips in the Kimura DGS curves (in dash curves) were twice or more of the present results at the same **S**. From these comparisons, the two present approaches and Kimura’s produced large differences in the near-field region. Possible causes cannot be ascertained at present due to the lack of knowledge of the Kimura method. Since the Kimura DGS is based on the Lommel formula, it is independent of **a_T_/λ**, as was the case for the Torikai DGS diagram, sharing the same drawback as the latter.

These comparisons demonstrate that all three published DGS diagrams have flaws and can only serve as an approximation. Among the three, the general DGS diagram had the most serious flaw due to the lack of transparency regarding its construction.

Finally, cases of non-circular transducers are briefly addressed. Kleinert [5,48] treated this topic for rectangular transducers and phased arrays. The latter consisted of square or rectangular elements. His work used an extension of the DGS calculation for a circular element, but key details were absent and provided minimal DGS diagrams. The DGS for the sound beams of a phased array includes the tilted transducer face and refractive interfaces, which make diffraction analysis intractable. Kleinert handled this part with a geometrical approach. While refractive interface problems were treated in [53], diffraction analysis for non-circular cases is too complex, making physics-based DGS diagrams elusive when the beam direction is off-axis. The MATLAB software used in this study [28] can calculate the sound fields of a rectangular transducer on the basis on San Emeterio and Ullate [54]. However, dealing with two-way diffraction requires **P–Z** relations between the rectangular transducer and a typically circular flaw. This is another difficult problem, even when using elaborate software. Thus, the DGS diagrams for non-circular transducers appear to be unattainable by calculations. On the other hand, practical DGS diagrams should be achievable using the experimental procedures of Krautkramer [36,37].

## 5. Summary and Conclusions

This study was conducted to quantitatively evaluate the sound fields of a circular ultrasonic transducer and to determine the DGS diagrams. Our findings are summarized and our conclusions presented.

1. Using the Torikai equations derived from the Lommel formulas and a MATLAB code based on the Zemanek model, the sound fields of a circular ultrasonic transmitter were determined quantitatively, producing a tabular listing of the sound pressure, **p**, vs. radial position, **X**, and propagation distance, **Z**. These were also integrated section-wise to the radial integration limit, **X′**, providing integrated sound pressures, **P**.
2. Between the two methods, general trends of the variation in **p** and **P** with respect to **X**, **X′**, and **Z** matched well in the far-field region, but their behaviors in the near-field region agreed only approximately. These were dependent on the calculation method as well as the ratio of the transmitter radius to wavelength, **a_T_/λ**. The Torikai equations were independent of **a_T_/λ**, while the Zemanek model yielded **a_T_/λ**-dependent **p** and **P** results.
3. In order to determine the DGS diagrams based on the two-way diffraction method, datasets of **P** vs. **Z** were separately obtained for the forward and backward paths since the backward path has a vastly different near-field distance. These two were combined on the basis of the propagation distance, and DGS diagrams were obtained. Six such DGS diagrams were presented and discussed. Similar to the sound pressure results, the DGS diagrams had general accordance, but differed in the near-field region.
4. The Torikai-based DGS diagram agreed well with the Zemanek-based one that used a 19 mm transmitter radius. Both showed a sharp dip at **Z** = 1 for smaller reflector sizes. The Zemanek-based DGS diagram for the smaller transmitter radii had a corresponding dip at lower **Z**, and eventually no dip. Thus, it is evident that multiple DGS diagrams need to be prepared to accommodate various ultrasonic test conditions.
5. Using the knowledge on the sound fields of a circular ultrasonic transducer, the published DGS diagrams were examined. The Mundry DGS diagram was found to agree with an approximate Torikai-based quasi-DGS diagram, which simplified the backward diffraction, assuming an equal-sized transmitter–receiver pair. This agreement also implied that Mundry utilized the Lommel formulas in his sound field calculations because of the matching position of the dips as well as the independence on **a_T_/λ**. The general DGS diagram was found to agree with the Mundry DGS diagram, except for the dips that were smoothed out. This simplified diagram cannot be treated as science-based since no justification had been presented for the curve alteration. The Kimura DGS diagrams matched the Torikai diagram reasonably well since Kimura also used the Torikai equations. However, arbitrary procedures were included, making one type of the Kimura diagram inappropriate.

The conclusions of this study are as follows:

1. Sound field calculations can be used to obtain DGS diagrams using a two-way diffraction method and proper accounting of the backward diffraction paths. Details of this construction method are presented. Two methods, Torikai and Zemanek, were used to obtain the sound fields. After a comparison of the results, the Zemanek-based calculations were found to properly account for the transmitter sizes. The ranges of the variables were still limited, and enlarged computations are needed to complete practical DGS diagrams. Other calculation methods by Stepanishen, Hasegawa et al. and Mast [23,24,25,26] should also be explored.
2. Three published DGS diagrams were evaluated and found to contain undisclosed approximations, making them unreliable for their general use. The general DGS diagram from ISO 16811 had the most technically serious simplifications by arbitrarily removing large variations in the Mundry DGS curves, from which the diagram was apparently constructed.

## Figures and Tables

**Table 1:** List of the parameters and symbols used.

	Forward Path	Backward Path
Transmitter size (Tx)	**a_T_**	**a_T’_** (=**a_R_**)
Receiver size (Rx)	**a_R_** (=**a_T_ S**)	**a_R’_** (=**a_T_**)
Ratio of Rx to Tx	**S** = **a_R_/a_T_**	**S_B_** = **1/S**
Near-field distance	**N** = **a_T_^2^/λ**	**N_B_** = **N S^2^**
Integrated sound field	**P**(**X′**,**Z**)	**P_B_**(**X′**,**Z_B_**)
Wave propagation distance	**Z**	**Z_B_**

## Data Availability

Not Applicable.
